# Detection and management of coal seam outcrop fire in China: a case study

**DOI:** 10.1038/s41598-024-55304-1

**Published:** 2024-02-26

**Authors:** Yang Liu, Xuyao Qi, Dayong Luo, Yongqing Zhang, Jiangtao Qin

**Affiliations:** 1 School of Resources and Security, Chongqing Vocational Institute of Engineering, Chongqing, 402260 China; 2 School of Safety Engineering, China University of Mining and Technology, Xuzhou, 221000 China

**Keywords:** Outcrop fire, Environment protection, Fire detection, Inversion interpretation, Hidden fire, Environmental sciences, Energy science and technology, Engineering

## Abstract

The outcrop fire area in Rujigou Coal Mine in Ningxia, China has been burning continuously for over 100 years. This not only results in wastage of resources but also poses significant damage to the ecological environment. Previous research on open fire detection has mainly focused on coalfield fire areas, using single method such as infrared remote sensing or surface temperature measurement, magnetic method, electrical method, radon measurement and mercurimetry. However, the outcrop fire area has migrated to deeper parts over the years, conventional single fire zone detection methods are not capable of accurately detecting the extent of the fire zone, inversion interpretation is faced with the problem of many solutions. In fire management, current research focuses on the development of new materials, such as fly ash gel, sodium silicate gel, etc., However, it is often difficult to quickly extinguish outcrop fire areas with a single technique. Considering this status quo, unmanned aerial vehicle (UAV) infrared thermal imaging was employed to initially detect the scope of the outcrop fire area, and then both the spontaneous potential and directional drilling methods were adopted for further scope detection in pursuit of more accurate results. In addition, an applicable fire prevention and extinguishing system was constructed, in which three-phase foam was injected for the purpose of absorbing heat and cooling. Furthermore, the composite colloid was used to plug air leakage channels, and loess was backfilled to avoid re-combustion. The comprehensive detection and control technologies proposed in this study can be applied to eliminating the outcrop fire area and protecting the environment. This study can provide guidance and reference for the treatment of other outcrop fire areas.

## Introduction

**Figure 1:**
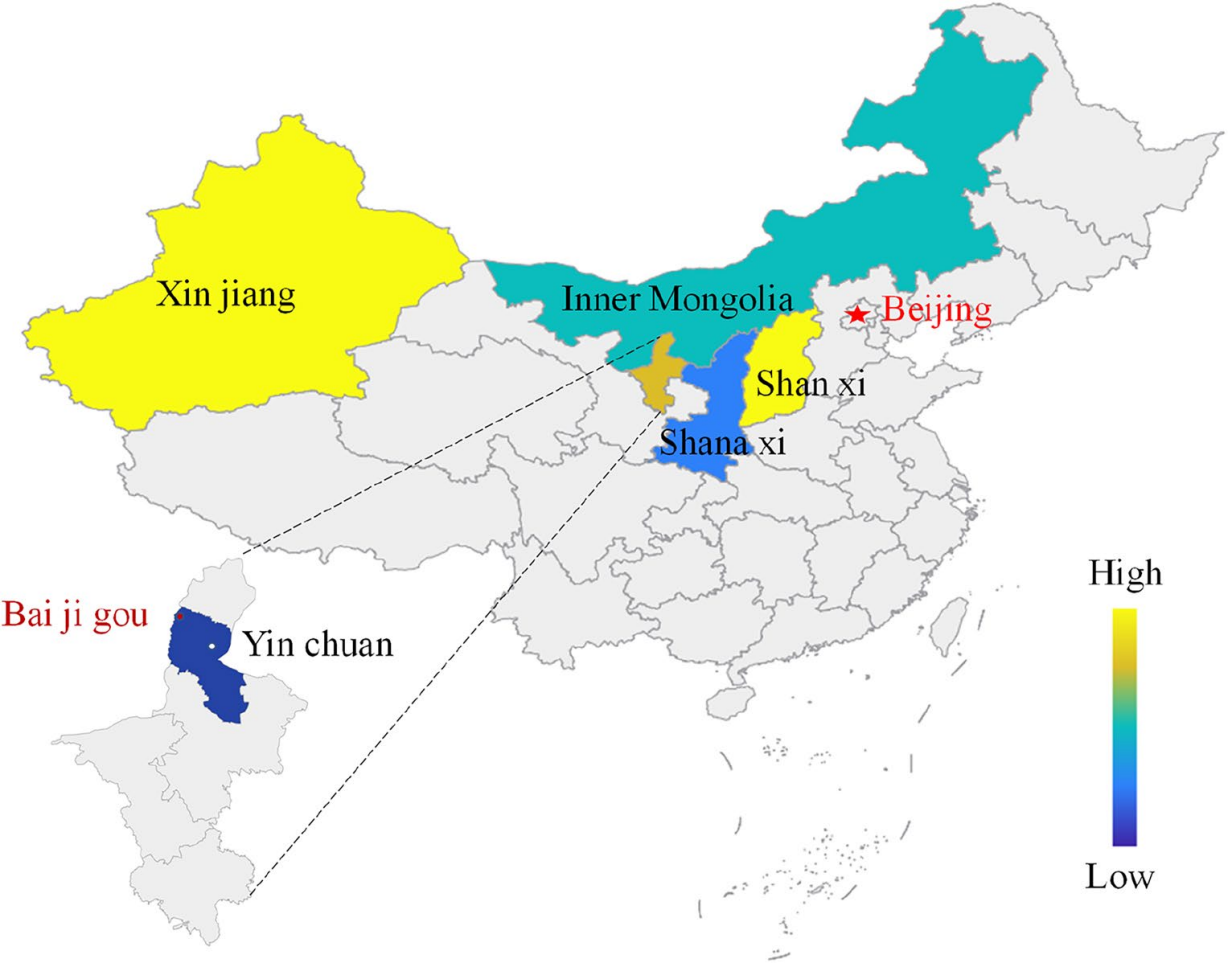
Geographical location map of the outcrop fire area in Baijigou Coal Mine (By EDRAW 13.0.1 https://www.edrawmax.cn).

Coal seam outcrop fire areas, if left uncontrolled, would lead to large-scale coalfield fires which seriously pollute the natural environment, cause casualties and threaten coal mine safety^1^. Coalfield fires occur in many countries around the world, such as the United States, India, South Africa and China, inducing global disasters^2,3^. Coalfield fires in the United States mainly hit areas such as Montana, Arizona, Wyoming, Colorado and Pennsylvania^4^. In India, coalfield fires mainly take place in Jharkhand, and in South Africa, Witbank and Sasolburg are at great risk^5,6^. As for China, the primarily affected region is northwest China which suffers from a lack of rain, including Inner Mongolia, Ningxia, Xinjiang, Shaanxi and Shanxi. Among them, Xinjiang and Shaanxi are the most seriously impacted, followed by Ningxia. Specific geographical locations are shown in Fig. 1^7^. The Xinjiang coalfield fire area ranks No. 1 in the world in terms of its area, and it has been burning for nearly a thousand years. According to the results of the fifth coalfield fire area survey in Xinjiang, the 40 untreated fire areas in Xinjiang cover a total area of 4.7773 million m^2^ by the end of 2019^8^. The Rujigou mining area in Ningxia, one of the most serious coalfield fire areas, has been burning for more than 100 years since the Qing Dynasty. According to statistics, the Rujigou fire area releases about 15,000 tons of particulate matter and 6000 tons of sulfur dioxide annually. Under the above circumstances, numerous scholars all over the world have engaged in research concerning coal seam outcrop fires.

Methods for detecting coalfield fire areas can be roughly classified into infrared remote sensing or surface temperature measurement, magnetic method, electrical method, radon measurement and mercurimetry^9^. In May 1963, HRB-Singer Company of the United States used the airborne thermal camera RECONOFAX infrared detection system to study coal gangue coal fires. This approach marks the beginning of applying the thermal infrared remote sensing technology to detect coal fires. Furthermore, this technology proves to be feasible through experimental analysis and comparison^10^. Aiming at easing open-pit mine management, Saini combined thermal infrared and visible light images captured by drones to quickly detect and accurately locate the thermal anomaly level of open-pit mining areas^11^. Mishra et al. took advantage of the thermal imaging and remote sensing technology to detect the fire area of the Jharia coalfield in India, and verified the effectiveness of this technology^12^. Chatterjee et al. studied the coal fire dynamics of the Jharia coalfield during the 1990s based on medium-resolution satellite thermal IR data such as Landsat-5 TM and Landsat-7 ETM + data (acquired in 10.4–12.5 µm spectral region)^13^. Guha et al. employed an advanced spaceborne thermal emission and reflection radiometer to map the latest distribution of coal fires in the Raniganj coalfield. The coalfire map shows that major fires are associated with open-pit mines of the coalfield^14^. With the widespread application of UAV, it is gradually known to the public that UAV remote sensing boasts the advantages of fast data acquisition and high measurement accuracy, providing a new idea for coalfield fire detection^15^.

Magnetic detection is mainly based on the principle that changes in magnetism can reflect how temperature varies because the magnetism of rock increases with the rise of temperature^16^. Lindqvist et al. detected a total magnetic field intensity of up to 8 A/m on the coal seam of burning lignite^17^. Ide et al. successfully found a way to divide the burned and unburned coal seams for South Ute using the magnetic method^18^. Kong et al. proposed an innovative method of applying the electromagnetic radiation technology to detect high-temperature anomaly areas in the Daquanhu fire area^19^. Vaish and Pal processed data on magnetism and investigated the magnetic field anomaly of East Basuria Colliery in the Jharia coalfield in Jharkhand, India, with a view to mapping and studying sub-surface coal fires. Additionally, they calculated the residual component of the total magnetic field anomaly map of the area, so as to enhance and delineate the coal fire area^20^.

Apart from the above methods, the electrical method, which mainly includes the spontaneous potential method and the electromagnetic method, also sees extensive application in the detection of coalfield fire areas^21,22^. Powel is the first to discover that the electrical conductivity of carbon changes with temperature under conditions of high temperature^23^. The spontaneous potential method has been successfully applied to a shallow coal seam fire in Colorado^24^. Shao et al. conducted sandbox experiments to invert the law of potential variation in coalfield fire areas, and further verified the effectiveness of spontaneous potential detection in coalfield fire areas^25,26^. Shao et al. wielded the magnetic and spontaneous potential methods to delineate the extent of the fire, and proposed a composite indicator to better indicate the fire. What’s more, the spontaneous potential method has been included in the “China Coal Fire Extinguishing Regulations”^27^.

Cheng et al. exercised the isotopic radon measurement technology to detect the coal seam outcrop fire areas in Liujiamao Coal Mine and Qian’an Coal Mine^28^. Du et al. proposed a comprehensive detection and verification method for high-temperature anomaly areas using a combination of radon level, infrared imaging technology and drilling method. The test results revealed that the radon content is positively correlated with the potential temperature^29^. Sun measured concentrations and isotope compositions of atmospheric Hg in both gaseous and particulate phases on an urban site near the Wuda coalfield^30^. The measurement results demonstrated that atmospheric Hg isotope measurement functions as a useful tool for detecting concealed underground coal fires. On this basis, the isotope radon measurement technology and mercurimetry are extensively applied to coalfield fire detection^31^.

In the treatment of outcrop fire areas, techniques such as stripping, yellow mud injection, inert gas injection and loess covering are generally adopted^32–35^. Shao et al. used the three-phase foam and water mist technology to deal with the upper fires in abandoned roadways and loose areas and to control the coalfield fires in Anjialing open-pit mine in China^36^. Tan et al. used sodium silicate gel, a new type of pressurized moisturizing plastic sealing material, to achieve rapid cooling of the fire area and extinguish the Fukang coal fire in Xinjiang, China^37^. Zhai and Deng introduced a new type of firefighting composite gel in response to outcrop fires, and described its features, performances and applications in detail^38^. Bustamante et al. discovered that diluted bitumen and brine (combustion inhibitor), cement/slaked lime, fine sand cement and clinker/slaked lime have application prospects in preventing fires. It was also determined that bitumen/brine enjoys superior adhesion and durability properties in the coal seam^39^. Deng et al. applied composite fly ash gel to Haibaoqing coal fires^40^.

Most of the above researches involved only a single outcrop fire detection method, including infrared remote sensing, surface temperature measurement, magnetic method, electrical method, or radon measurement. Since each detection method has its own advantages and disadvantages, it is hard to accurately identify the scope of the fire area using only one of them. Besides, considering varying merits of different methods, merely using a single fire prevention and extinguishing method may not only fail to treat outcrop fire areas efficiently, but also result in resource waste^41,42^. Therefore, technologies regarding precise detection and economical and efficient fire prevention and extinguishing for outcrop fire areas need to be studied and put into practice urgently.

In this study, with the southern outcrop fire area in Baijigou Coal Mine as the research object, the outcrop concealed fire area was detected and treated with the multiple advanced technologies. Based on the practice, a set of effective methods and technologies were summarized. The research findings are expected to provide a reference for the detection and treatment of coal seam outcrop fire areas.

## Overview of the fire area

**Figure 2:**
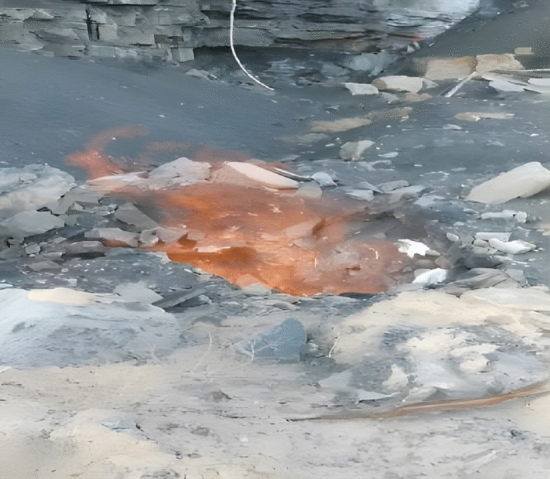
Fire in the outcrop fire area before treatment.

Baijigou Coal Mine is located in the northern part of the Rujigou mining area in the middle of the Helan Mountain. It belongs to the Ningxia Coal Industry Co., Ltd. of the National Energy Group. From the aspect of administrative division, it is under the jurisdiction of the Dawukou District of Shizuishan City, Ningxia Hui Autonomous Region, China. The central geographical coordinates are 106° 08′ 00′′ E and 39° 06′ 00′′ N, and its geographical location is given in Fig. 1. As depicted in Fig. 2, the outcrop fire burns along the coal seam line, giving rise to a raging fire, which not only wastes valuable coal resources, but also deteriorates the environment.

**Figure 3:**
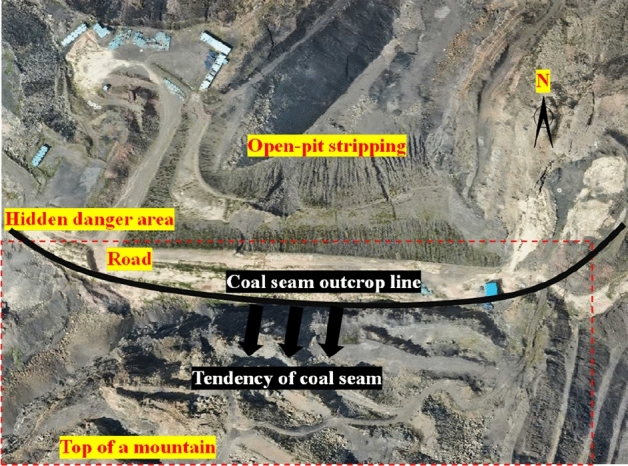
Overall location of the southern outcrop fire area (By Pix4dmapper 4.8.4 https://pix4d.com.cn/pix4dmapper).

Baijigou Coal Mine is attributed to a high gas mine. The underground 010,203 working face is the face to be mined, and the southern outcrop fire area exists near the surface above it. The surface southern outcrop fire is burning at the II_3_ coal seam which is distributed in a strip. The coal seam outcrop line in the fire area is about 290 m long and 70 m wide at most. Its north side lies an open-air stripping pit, and the south side is the original mountain. The southern outcrop fire area took shape as a result of open-pit stripping. During the open-pit stripping process, signs of combustion were found near the outcrop of the southern mining area. The overall location of the southern outcrop fire area is exhibited in Fig. 3.

**Figure 4:**
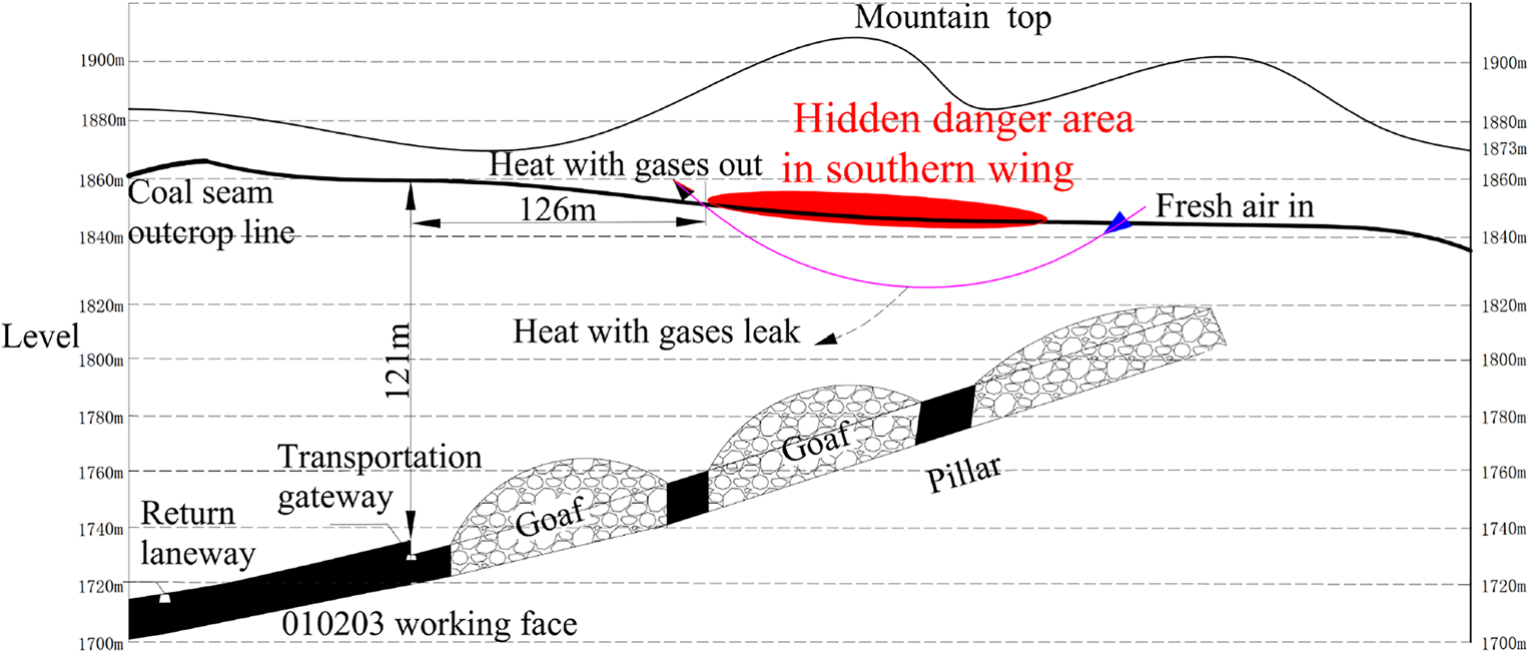
Sectional drawing of the southern outcrop fire area.

The southern outcrop fire area is located on the west side of the 010,203 working face, the horizontal and vertical distances between them being 126 m and 121 m respectively. Under the influence of surface air leakage, the outcrop fire area gradually extends to deeper sites along the coal seam outcrop line, giving off a large quantity of open flames and toxic and harmful gases through surface fractures. At the same time, as the 010,203 working face advances, these toxic and harmful gases and high-temperature heat sources may migrate towards the 010,203 working face through leakage channels under the condition of underground full negative pressure ventilation. Worse still, these toxic and harmful gases and high-temperature heat sources, once encountering gas accumulated in the goaf, are likely to trigger gas explosion accidents and threaten underground coal mining operations (Fig. 4).

## Treatment scheme for the outcrop fire area

**Figure 5:**
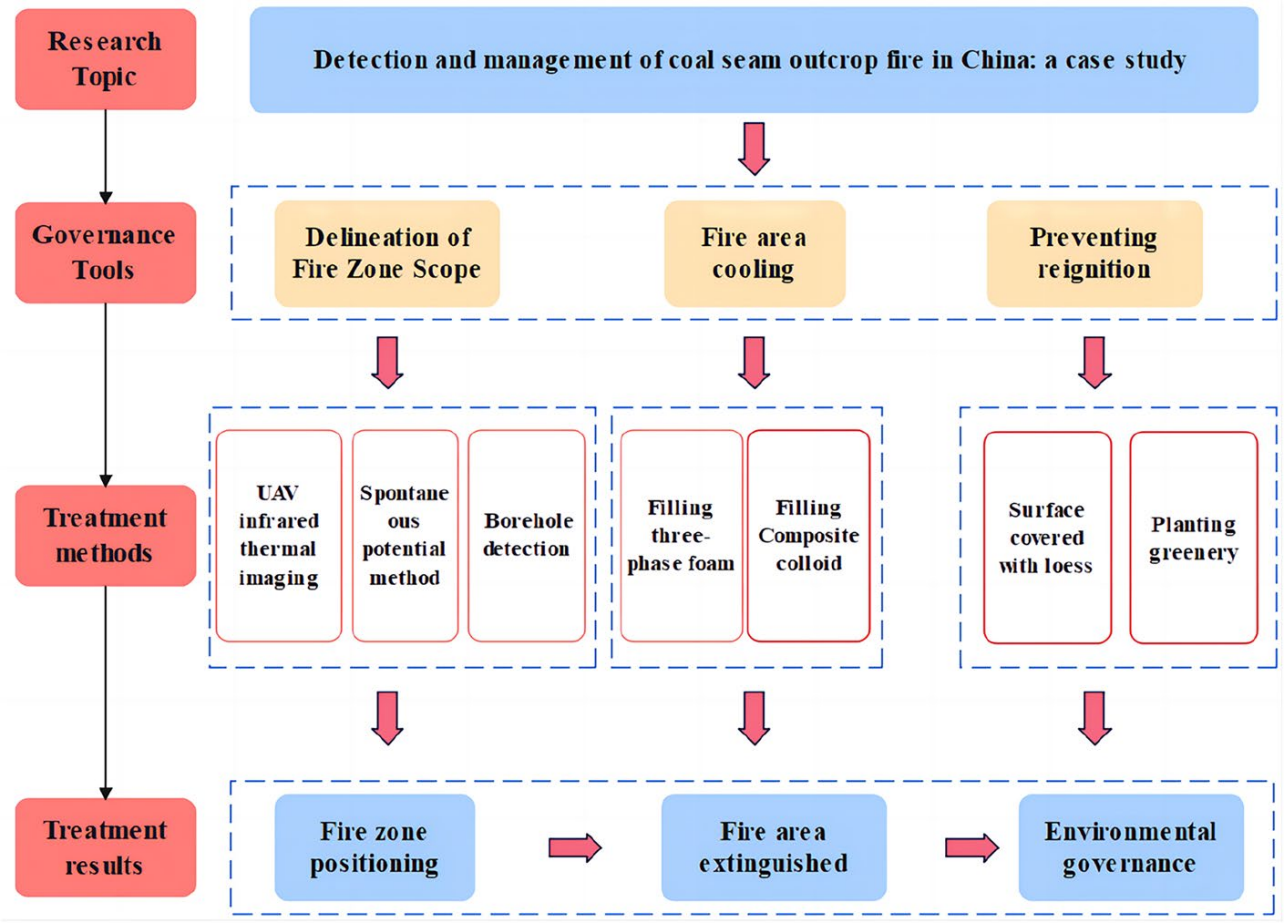
Treatment scheme for the coal seam outcrop fire area.

Referring to the characteristics of the outcrop fire area, a treatment scheme is formulated for it (Fig. 5). Firstly, the infrared UAV is used to conduct preliminary detection of the high-temperature area on the surface, and the suspected fire area is preliminarily determined. Subsequently, the spontaneous potential method with strong anti-interference ability is arranged on site to detect the hidden fire area and narrow the scope of the initially delineated fire area. Finally, boreholes are constructed across the seam. For one thing, the accurate range of the fire area can be determined. For another, these boreholes contribute to the following fire extinguishing. After the completion of borehole construction, the fire gas and temperature indicators in the boreholes are measured for evaluating the development of the outcrop fire area. Once the range of the outcrop fire area is identified, the next step is to inject three-phase foam to cool down the hidden fire area by virtue of its characteristics of diffusion and accumulation. In addition, composite colloid is also poured into in the hope of plugging air leakage channels. At last, the outcrop fire area is covered with loess to prevent re-combustion.

## Detection of the outcrop fire area

### Preliminary study on the surface based on UAV infrared thermal imaging

Infrared thermal imaging, a kind of passive infrared night vision technology, is based on the fact that all objects in nature with temperatures higher than absolute zero (− 273 °C) radiate infrared at all times. Such infrared radiation carries information concerning characteristics of the object, which provides an objective basis for using the infrared technology to distinguish the temperature and thermal distribution fields of various measured targets. According to the above principle, the photoelectric infrared detector converts the power signal radiated by the heating part of the object into an electrical signal, and the imaging device can simulate the spatial distribution of the surface temperature of the object precisely. Finally, the simulation results are processed and the thermal image video signal comes into being, which is then transmitted to the display screen. Thus, the thermal image corresponding to the thermal distribution of the object surface is obtained, that is, the infrared thermal image. The UAV thermal imager is a well-performed infrared-based imager that can be controlled remotely, and it can detect high-temperature areas that are inaccessible to humans. In the light of this advantage, the M300RTK UAV thermal imager is first used to detect the surface area on the south side, so as to have a rudimentary grasp of the outcrop fire area. The M300RTK UAV boasts favorable performance and can support the ZenmuseH20 hybrid sensor that is composed of a laser rangefinder, a zoom camera, a wide-angle camera and a thermal imaging camera. With this equipment, the high-temperature area of the surface fire area can be accurately and rapidly detected.

Through online task recording, the thermal imager of the M300 RTK UAV is able to record various actions such as aircraft movement, pan-tilt pitch, photo shooting and zoom in real time. Furthermore, these recordings, if stored as route files, can be utilized at any time in future automatic inspection tasks. At the same time, it is equipped with an AI-assisted re-shoot function, which greatly improves the accuracy of automation tasks. After completing online task recording, the target area is selected from the sample frame. In subsequent automation tasks, the AI will automatically compare the target area with the current real-time picture, and correct the camera’s shooting angle accordingly, so that the same target area can be captured every time. Performances and parameters of the M300 RTK UAV are listed in Table 1.

**Table 1:** Performances and parameters of the M300 RTK UAV.

Performance	Parameter
Maximum flight altitude	5000 m
Maximum flight time	55 min
Maximum wind speed	15 m/s
Obstacle perception range	0.1–8 m
Service environment	Diffuse reflection; large size; high reflectivity (reflectivity > 10%)
Resolution	18,650 Li-ion (5000 mAh @ 7.2 V)
Frame rate	30 fps

**Figure 6:**
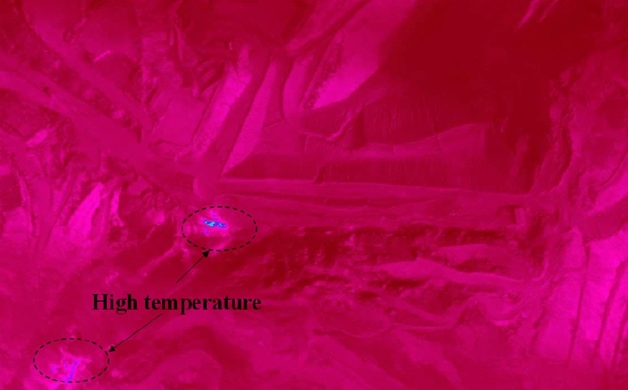
Cloud map of infrared thermal imaging.

**Figure 7:**
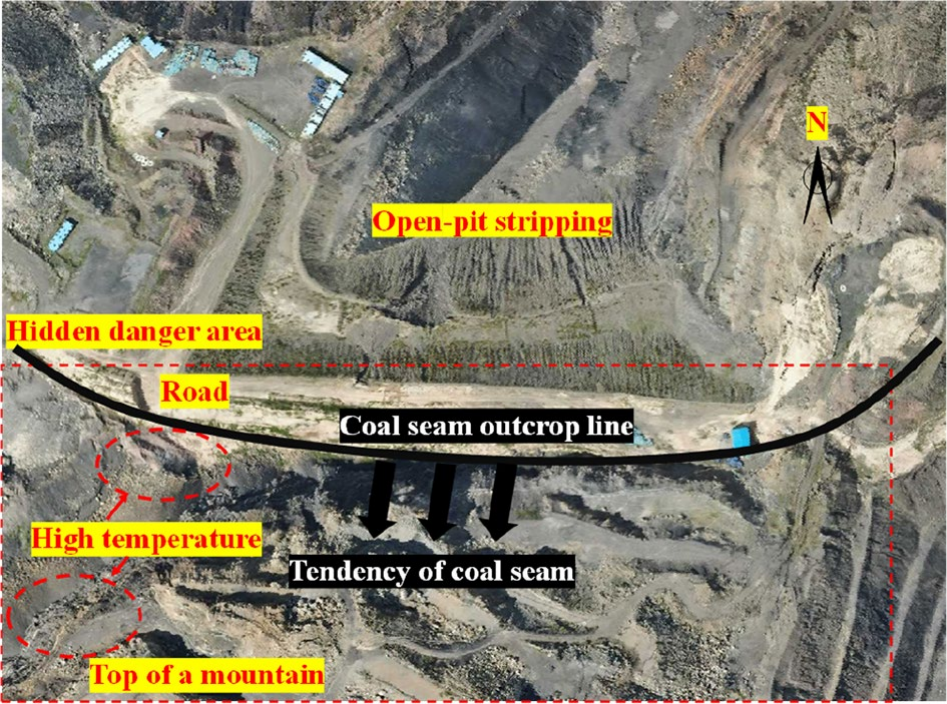
Surface of high-temperature areas (By Pix4dmapper 4.8.4 https://pix4d.com.cn/pix4dmapper).

On September 1, 2021, two UAV flights were carried out on the southern fire area of Baijigou Coal Mine in an effort to collect topography and infrared thermal imaging data. During the test, the relevant parameters were set as follows: the outdoor temperature 18 °C, the relative humidity 70%, no sustained wind, wind strength below Level 3, the flight height of the UAV 200 m, heading overlap rate 90% and side overlap rate 90%. Besides, the parameters of the M300 RTK infrared system and the camera were adjusted accordingly, and they adopted the high gain mode. The temperature curve was set to TIFF. Under these parameters, the generated visible light numerical orthophoto with a resolution of 0.2 m is exhibited in Fig. 7, and the detected cloud map of surface temperature imaging with a resolution of 0.4 m is shown in Fig. 6.

**Figure 8:**
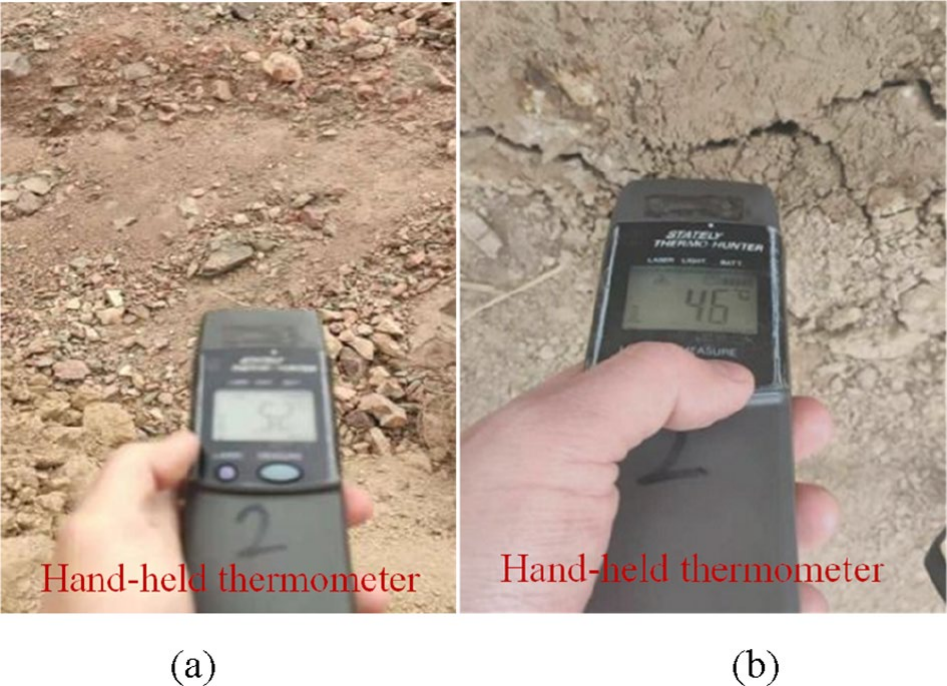
Temperature measurement by hand-held thermometer. (**a**) High-temperature Point A, (**b**) High-temperature Point B.

On the surface of the southern outcrop fire area, two high-temperature areas were detected. These two areas were concentrated in the slag platform on the west side (a maximum temperature of 70 °C) and in the valley on the southwest side (a maximum temperature of 65 °C). The surface of the high-temperature areas is drawn in Fig. 7. Later on, a hand-held infrared thermometer was used for re-testing, and the temperatures of the slag platform and the valley were 52 °C and 46 °C, respectively (Fig. 8). Further analysis reveals that although the high temperature was detected only in the west slag platform and the southwest valley, it does not mean that the outcrop fire area only existed on the surface. As coal spontaneous combustion intensified, chances are that the fire would invade the deeper part of the mountain along the outcrop line of the coal seam. The high temperature tracked in the southwest valley is a case in point.

### Delineation of the fire area based on the spontaneous potential method

**Figure 9:**
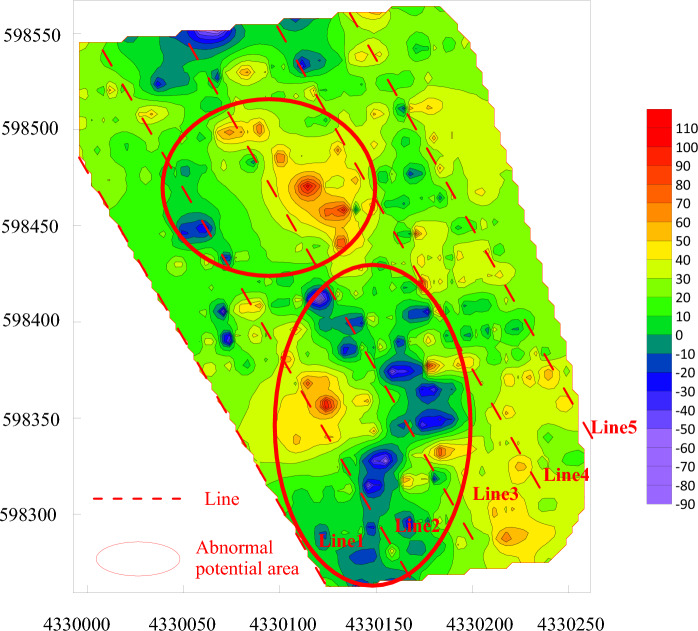
Contour of spontaneous potential anomaly areas in the outcrop fire area.

**Figure 10:**
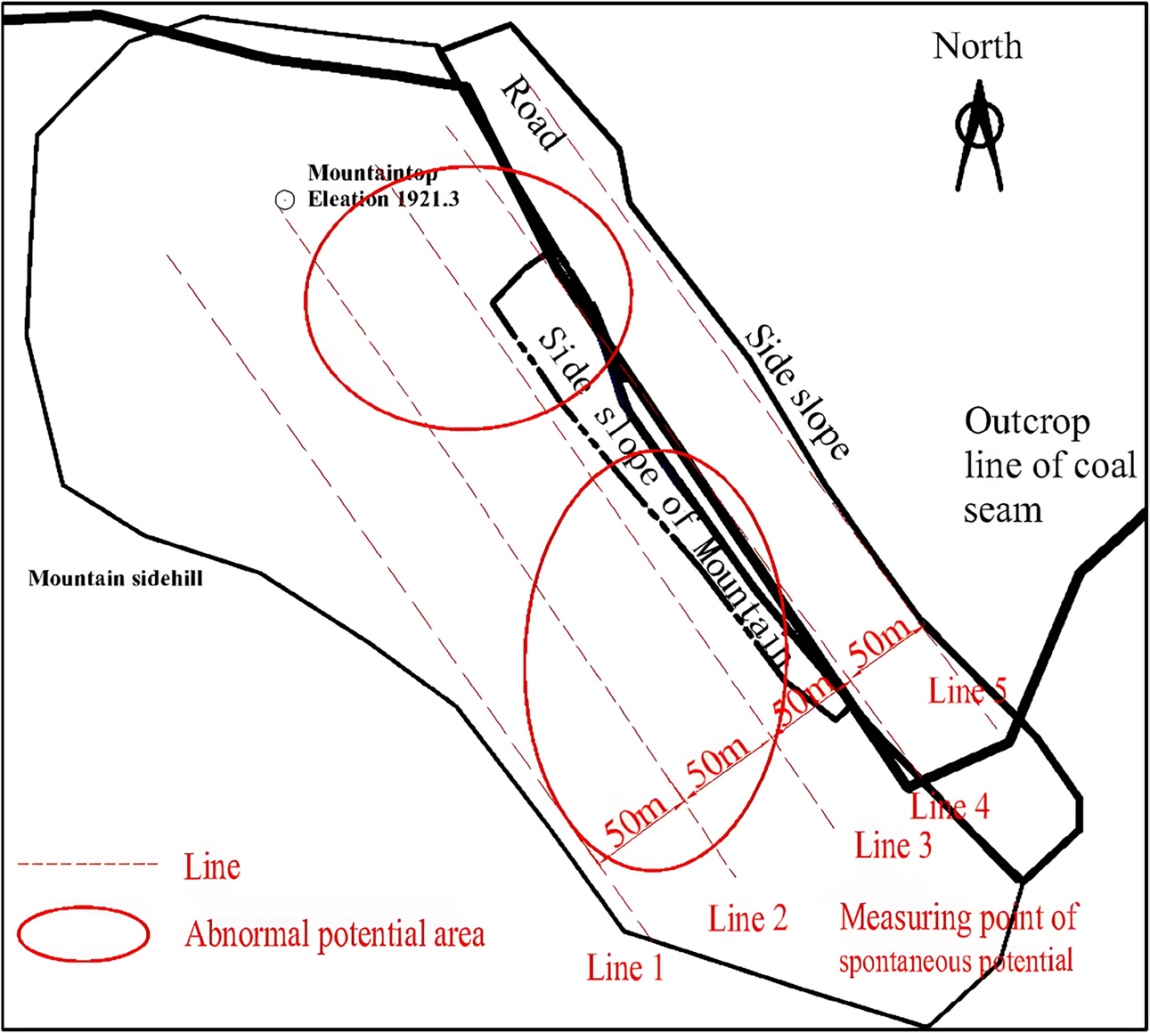
Comparison diagram of spontaneous potential anomaly areas in the outcrop fire area.

In view of the fact that UAV infrared thermal imaging is limited to high-temperature detection on the shallow surface (< 10 m), it is generally used for the early detection of the outcrop fire area, and it fails to determine the location of the hidden fire source in the outcrop fire area. The authors make a comparison among current feasible fire detection methods, such as isotope radon measurement, magnetic method and spontaneous potential method. Among them, isotope radon measurement is susceptible to the properties of overlying strata, fractures, water content and weather. Besides, it corresponds to poor accuracy and weak anti-interference ability. The magnetic method proves to enjoy a good response to the burnt rock after the fire. Despite this, it cannot timely react to the high-temperature fire area that is burning. Consequently, neither of them are suitable for the detection of the southern fire area of Baijigou Coal Mine^43^. Ultimately, the spontaneous potential method was selected to detect the fire area. The spontaneous potential method is a geophysical method primarily applied to the exploration of the distribution and anomaly of spontaneous potential. By detecting the anomaly of spontaneous potential, the range of the coalfield fire area could be deduced. Based on the actual situation of the site, five detection lines were arranged with a spacing of 50 m, and measuring points were located every 5 m. At last, a total of 640 sets of spontaneous potential data were collected using a WDJD-1 multifunctional digital DC IP instrument and a pair of Cu–CuSO_4_ non-polarizing electrode electrodes. The contour of the spontaneous potential anomaly area is drawn in Fig. 9. A detailed analysis on Fig. 9 indicates that positive potential anomaly areas exist in the coordinates such as [4330120, 598280]–[433020, 598430], [4330030, 598420]–[4330160, 598520] in the absence of cluttering areas. The preliminary delineation of the fire area is given in Fig. 10.

### Verification based on the drilling detection

The location of high-temperature areas was preliminarily determined after elementary detection by thermal imaging and anomaly area determination by spontaneous potential. In order to verify the exact scope of the fire area and lay the foundation for the later fire extinguishing project, further detection was carried out through the directional drilling from the surface to the outcrop coal seam. It is noteworthy that the drilling was timely adjusted according to the detection area. A total of 15 deep holes were drilled using a φ219 mm pipe-following drill bit towards a depth of 25 m (the slope is not drilled). The φ219 mm × 8 mm casing ran under the pipe, and the total amount of drilling was 1579 m. The layout of boreholes is depicted in Fig. 13.

A significant high-temperature area is observed near the northwest side of the 1#, 2#, 3# and 4# boreholes in the outcrop fire area. Along the northwest direction, the temperature increases remarkably, reaching the highest at the depth of 100 m.

**Figure 11:**
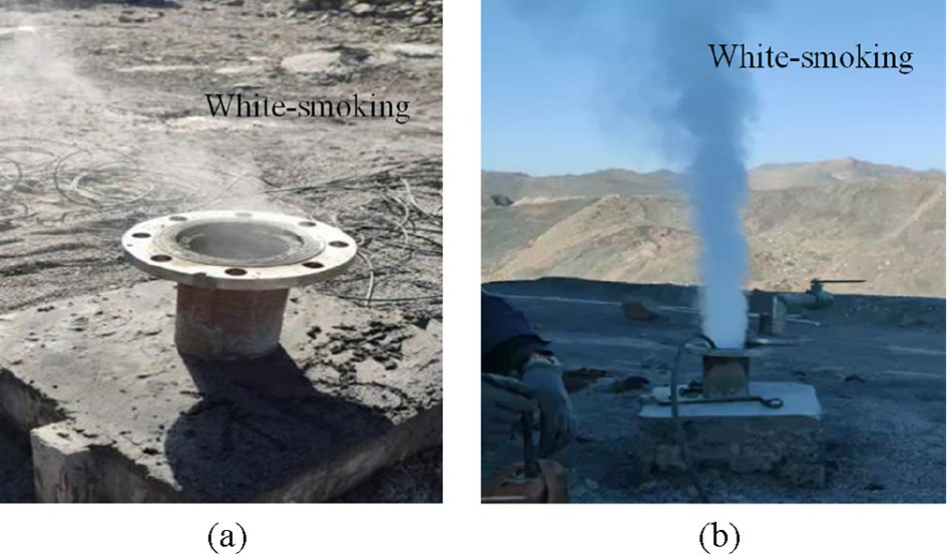
White smoke emitted from boreholes. (**a**) Borehole A, (**b**) Borehole B.

**Figure 12:**
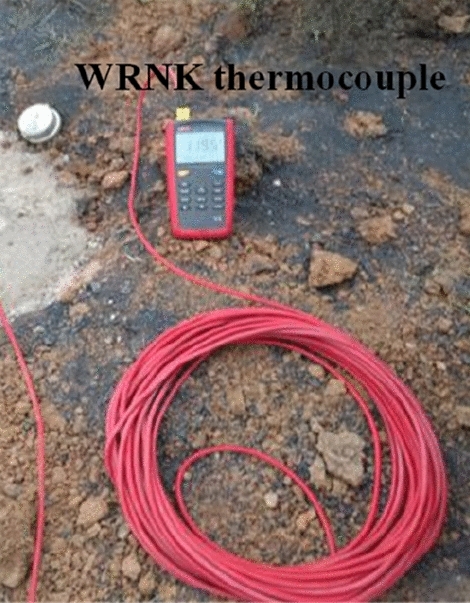
Temperature of the borehole measured by the WRNK thermocouple.

In addition, the spontaneous combustion indicator gases (carbon monoxide, ethylene and acetylene) of coal in this area are extremely prominent. At the same time, the phenomenon that a large amount of white smoke is emitted from the borehole is observed during the process of drilling (Fig. 11). A thermocouple is used to measure the temperature in the borehole, and it is found that the local temperature is as high as 260 °C, which indicates that the shallowly buried outcrop coal near the high-temperature anomaly area has shown signs of combustion. The temperature measurement results are displayed in Fig. 12. From Table 2, it is evident that the temperatures of 9#, 10#, 11#, 12# and 13# boreholes on the south side are rather high, slightly lower than that on the northwest side. Judging from this, it is credibly inferred that the outcrop coal has spread further along the coal seam outcrop line to the north side.

**Table 2:** Abnormal temperatures and spontaneous fire indicator gases of boreholes.

Borehole No	Temperature	CO (%)	CO_2_ (%)	C_2_H_6_ (ppm)	C_2_H_4_ (ppm)	C_2_H_2_ (ppm)
1	101	0.2756	9.58	99	1	0
2	140	0.8049	22.81	141	6	1
3	280	1.0007	22.5	491	3	0
4	90	0.1310	10.152	100	1	0
9	64	0.0519	2.2	5	0	0
10	58	0.0007	0.15	0	0	0
11	58	0.0001	0.01	1	0	0
12	50	0.0007	0.22	5	0	0
13	52	0	0.04	4	0	0

**Figure 13:**
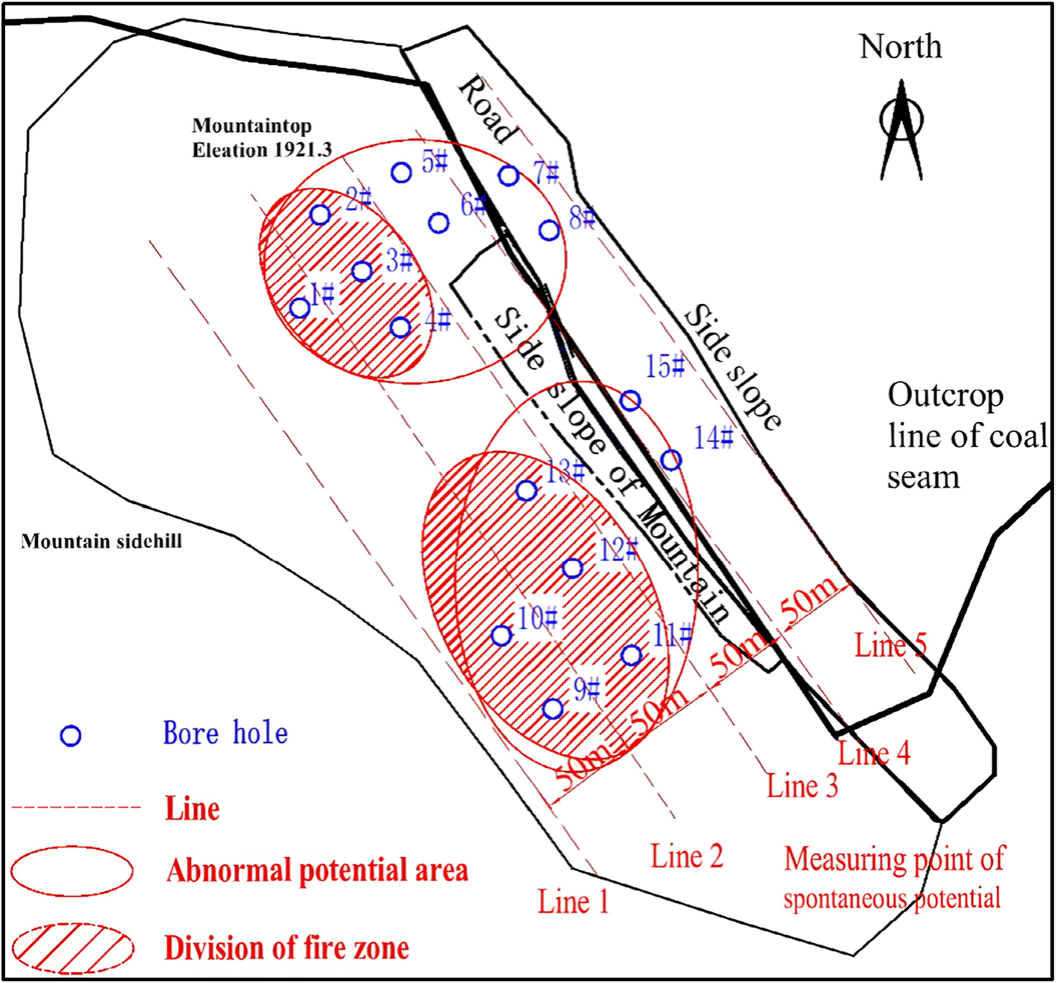
Potential fire area range of the southern outcrop fire area.

Temperature measurement was also conducted in the remaining mountain supplementary boreholes (5#, 6#, 7#, 8#, 14# and 15# ), and no obvious abnormal high temperature was captured. Although a trace of coal spontaneous combustion indicator gases were occasionally detected in these boreholes, they were possibly attributed to the harmful gases that intruded into the flow field from the fire area through fractures due to the development of mountain fractures and the complexity of air leakage channels. This indicates that the fire area has not spread to the east side near the working face. Based on this, the location of the southern outcrop fire area was determined, and the potential fire area range was delineated (Fig. 13).

## Comprehensive fire prevention and extinguishing technology

In accordance with the basic features of the outcrop fire area and the detection results of the fire area, the outcrop fire area has spread along the coal seam line to the mining area, and intensified from west to east. Additionally, the high-temperature fire source is deeply buried, and the high-temperature hazard area is large, concealed and three-dimensionally distributed, which makes it difficult for a single fire extinguishing technology to play a role. For this reason, it is of great necessity to build a comprehensive fire prevention and extinguishing system on site.

### Construction of comprehensive fire prevention and extinguishing system

**Figure 14:**
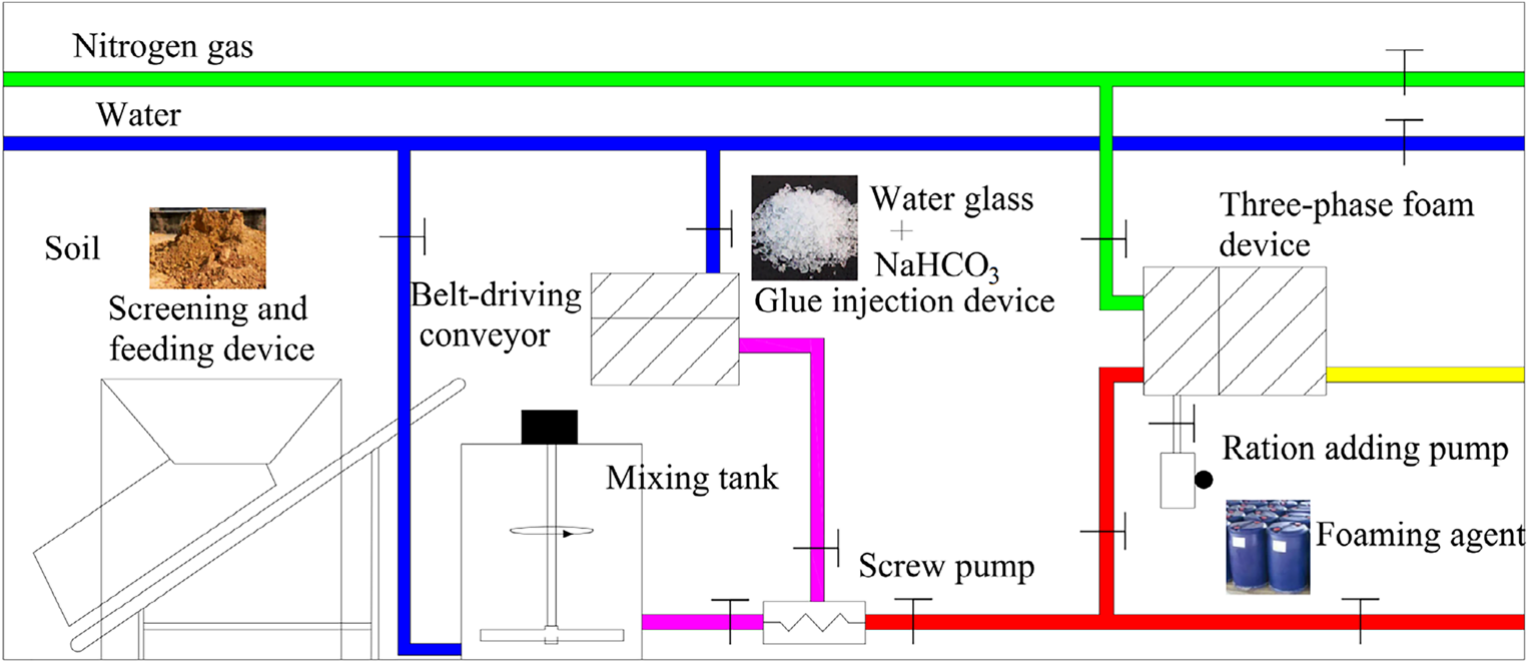
Comprehensive fire prevention and extinguishing system.

For the sake of achieving the four functions of water, grout, three-phase foam and composite colloid injections, a comprehensive fire prevention and extinguishing system was constructed according to local conditions (Fig. 14). The whole system is driven by a motor and furnished with a water supply pipeline and a nitrogen supply pipeline. The pressure of nitrogen should be higher than or equal to 0.5 MPa, and its purity is required to exceed 99.99%. As for the water supply, the pressure ought to be higher than 3 MPa, and the flow rate must surpass 20 m^3^/h. The concrete procedure for preparing composite colloid is as follows: firstly, the loess is loaded by a forklift for screening, and then transported to a mixing tank through an automatic feeding belt. After being mixed, the yellow mud is formed. Subsequently, the yellow mud, water glass and sodium bicarbonate are blended, during which the pressure is raised using a screw pump. In this way, the composite colloid is successfully prepared. In terms of the preparation of three-phase foam, it can be realized by mixing yellow mud and the foaming agent of nitrogen.

### Treatment of high-temperature area

#### Injection of three-phase foam

The three-phase foam technology integrates the functions of plugging, inerting, covering and inhibiting. High-expansion foam can quickly fill large tracts of fractures, and its favorable fluidity and covering ability can support fire extinguishing and cooling at hidden fire sources. The slurry from the mixing tank is transported into the pipeline through the filter screen, and 0.5–1% foaming agent is added to the slurry through the foaming agent quantitative addition pump. The slurry and the foaming agent are mixed evenly in the pipeline through the mixer before entering the foaming device. In the foaming device, nitrogen is injected and interacts with the slurry containing foaming agent to produce three-phase foam, which is later injected into the fire extinguishing boreholes through the shunt.

**Figure 15:**
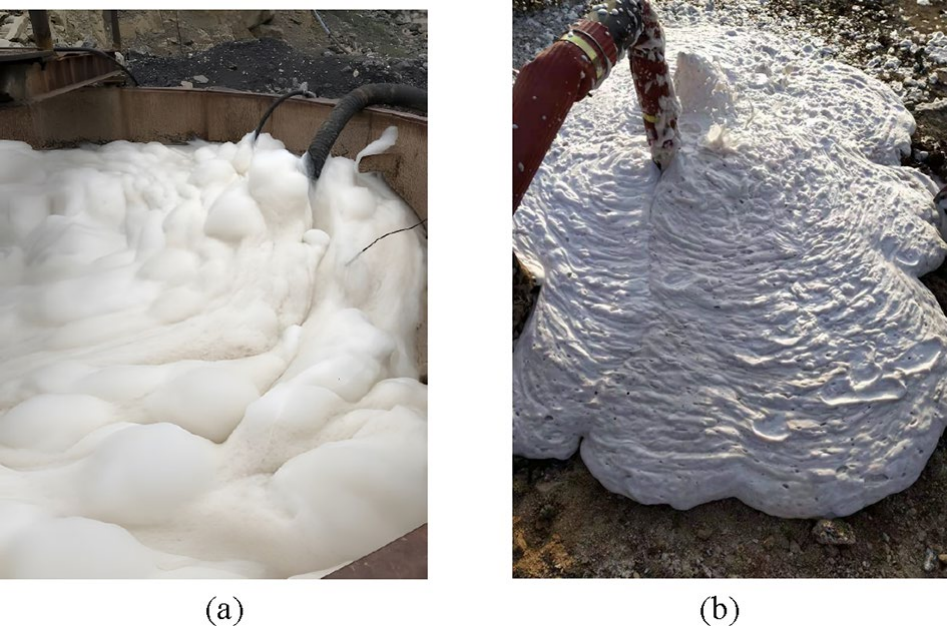
Pouring three-phase foam into boreholes: (**a**) preparing three-phase foam; (**b**) pouring three-phase foam.

According to the laboratory experimental research, field industrial test, as well as the expansion coefficient of coal and rock strata (1.4), the relevant parameters are set as follows: the addition ratio of the foaming agent 1%, the foam expansion 30 times, the foam stability time ≥ 8 h, a single borehole filled with 658 m^3^ yellow mud and 19,740 m^3^ three-phase foam, and the foaming agent 6.6 tons. The process of pouring three-phase foam into boreholes in the field is displayed in Fig. 15.

#### Injection of composite colloid for air leakage channel plugging

**Figure 16:**
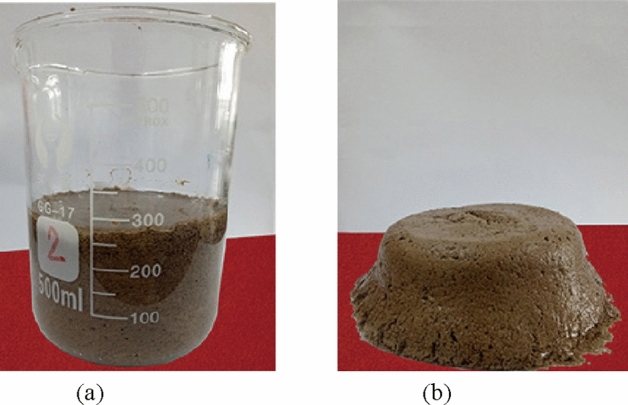
Coagulation effect of composite colloid prepared in the laboratory: (**a**) Water-retaining property after mixing composite colloid with yellow mud; (**b**) Flip the beaker upside down on the table to observe the sedimentation effect of yellow mud.

**Figure 17:**
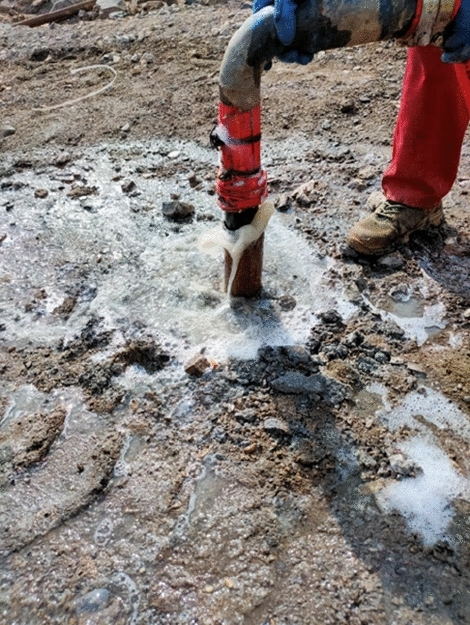
Filling boreholes with composite colloid in the field.

To prevent hidden high-temperature seedlings in the outcrop coal from re-combustion due to air leakage from fractures and boreholes, it is of urgent need to fill the boreholes and fractures that have met the requirements of cooling and fire extinguishing with water–glass–gel (NaHCO_3_ and water glass) in time. The composite colloid enjoys a high solid–liquid mass ratio of up to 1.5, which is 7 times that of pure yellow mud. Apart from this advantage, the composite colloid, if adsorbed with water and loess, can retain water for a long time and prolong the validity of the slurry. The sediments formed by hydrogel and loess features high solid content and good adhesion, which can effectively fill fractures and prevent the fire area from leakage-induced re-combustion. Figure 16 presents the coagulation effect of composite colloids prepared in the laboratory, and Fig. 17 illustrates the diagram of filling boreholes with composite colloid in the field.

In the hope of preparing high-quality and viscous composite colloid to achieve the optimal air leakage plugging and fire extinguishing effects, it is necessary to control the curing time of the colloid filling material by comprehensively combining the process flow, the required filling amount of fire extinguishing boreholes and the actual demands of fracture plugging and re-combustion prevention. Ultimately, the parameters are determined as follows: water-cement ratio 4: 1, the soda-sodium silicate-yellow mud ratio 1: 3: 20, each borehole filled with 6.6 tons of water glass and 2.3 tons of soda, and total filling space 54 m^3^.

### Treatment of medium- and low-temperature areas

Considering the treatment cost, medium and low-temperature boreholes (9#–13#) are cooled by injecting yellow mud. Yellow mud boasts favorable fluidity and plugging performance, which contributes to eliminating the hidden danger of medium and low-temperature boreholes. The flow rate of yellow mud is set to 30 m^3^/h, and the water-soil ratio is 3: 1. After the injection of yellow mud, the temperatures of boreholes need to be monitored in real time. If the temperatures rise dramatically, three-phase foam needs to be injected at once for cooling. Once the temperatures drop to normal temperature and there are no signs of a rebound, composite colloid is added to block air leakage channels and prevent re-combustion. The process and technical parameters of composite colloid injection in these boreholes are consistent with those in high-temperature boreholes, which will be not repeated here.

### Loess covering

**Figure 18:**
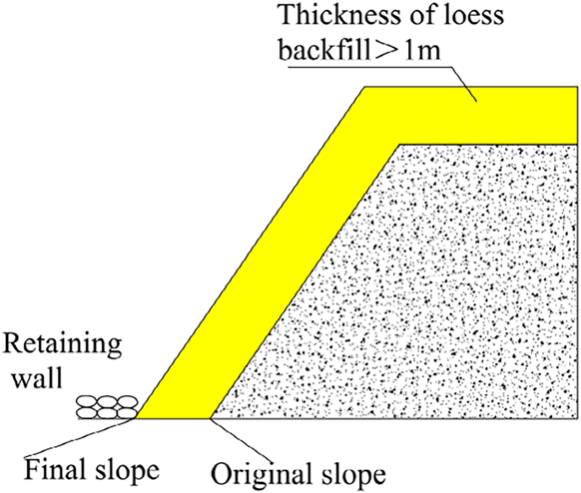
Loess backfilling on the surface and the slope.

**Figure 19:**
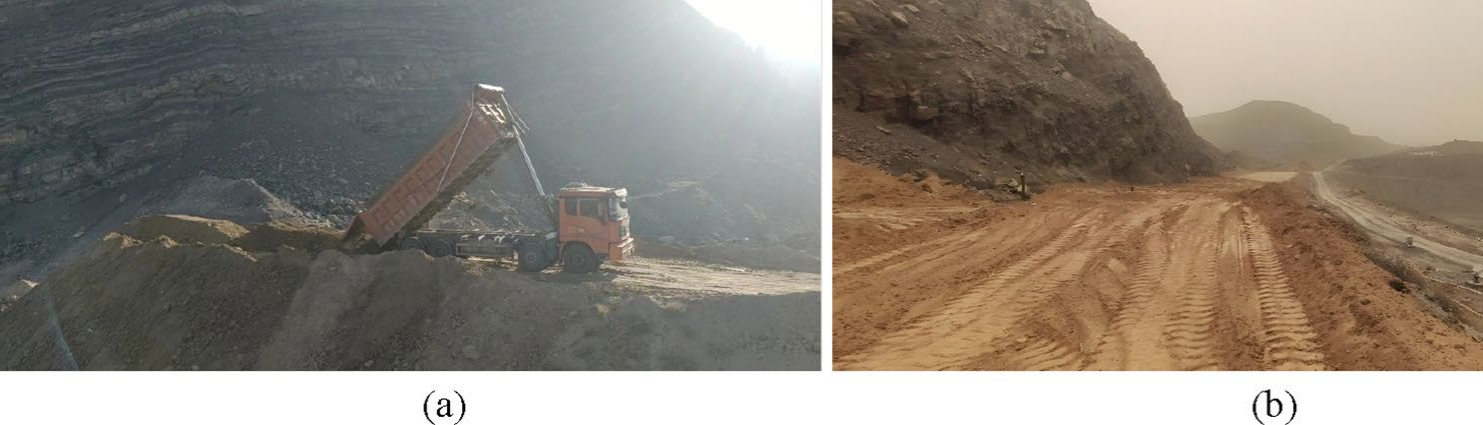
Loess backfilling on the slope: (**a**) Slope dumping of loess; (**b**) Slope top covering with loess.

After the southern outcrop fire area is cooled down and boreholes and fractures are filled, a retaining bag wall is stacked at the bottom of the northern slope. Next, the whole outcrop fire area and slope are backfilled and covered with loess. Following this treatment, a road roller is utilized to compact it immediately. The schematic diagram of loess backfilling is sketched in Fig. 18. The area of the southern outcrop fire area to be backfilled is about 35,000 m^2^ and the backfilling height will not be less than 1.0 m. Given the loose paving coefficient of 1.2, an earthwork volume of 40,000 m^3^ is used. The field operation can refer to Fig. 19.

## Discussion

In the early stage of treatment, spontaneous combustion in the southern outcrop fire area is severe. Through temperature measurement by means of drilling, it is detected that the temperatures of some boreholes in the delineated fire area reach up to 280 °C. The temperatures inside the boreholes are extremely high, so are the values of spontaneous combustion indicator gases such as carbon monoxide (up to 1 × 10^5^ ppm). Moreover, a large amount of water vapor and smoke are observed in the boreholes of the fire area, suggesting a fierce fire before treatment.

**Figure 20:**
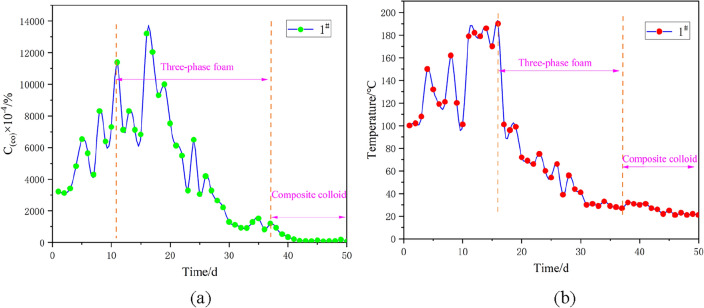
Treatment effect analysis for 1# borehole: (**a**) Variation of carbon monoxide concentration in the borehole with time; (**b**) Variation of temperature in the borehole with time.

**Figure 21:**
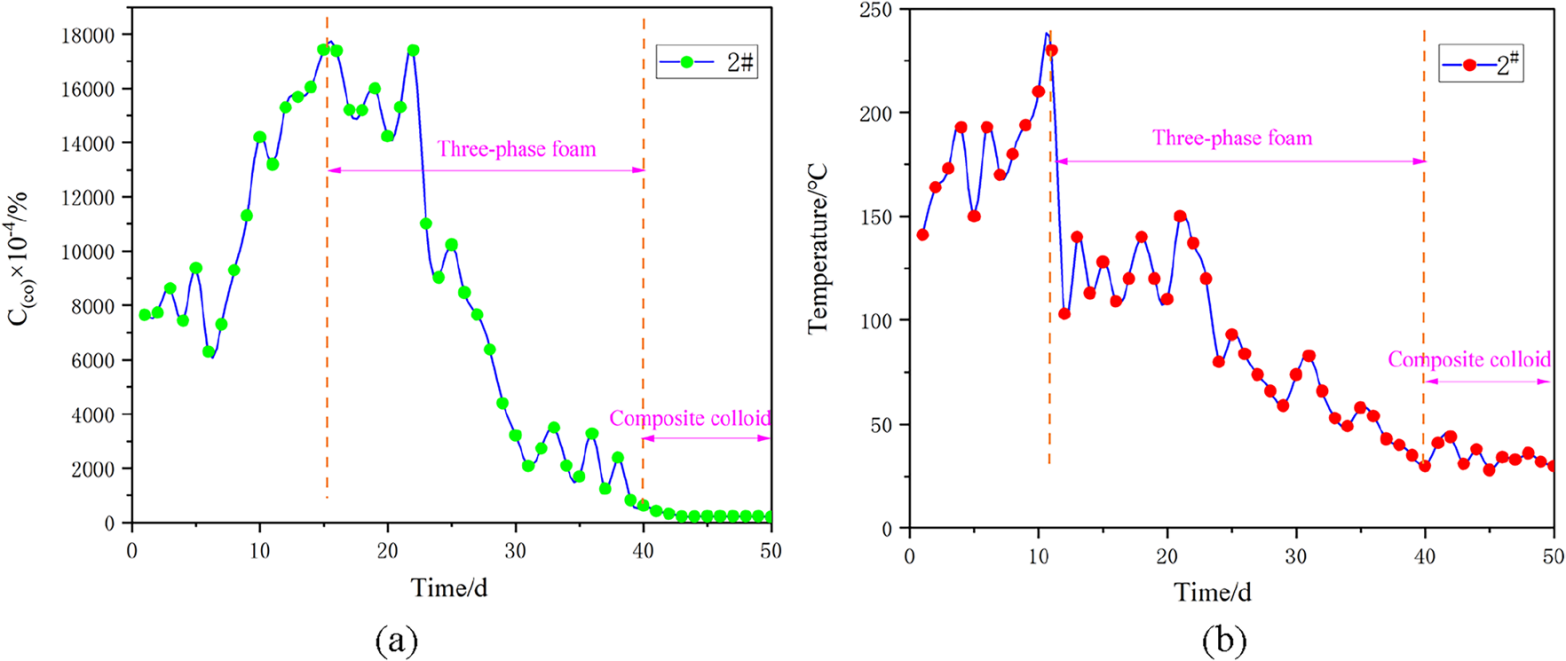
Treatment effect analysis for 2# borehole: (**a**) Variation of carbon monoxide concentration in the borehole with time; (**b**) Variation of temperature in the borehole with time.

**Figure 22:**
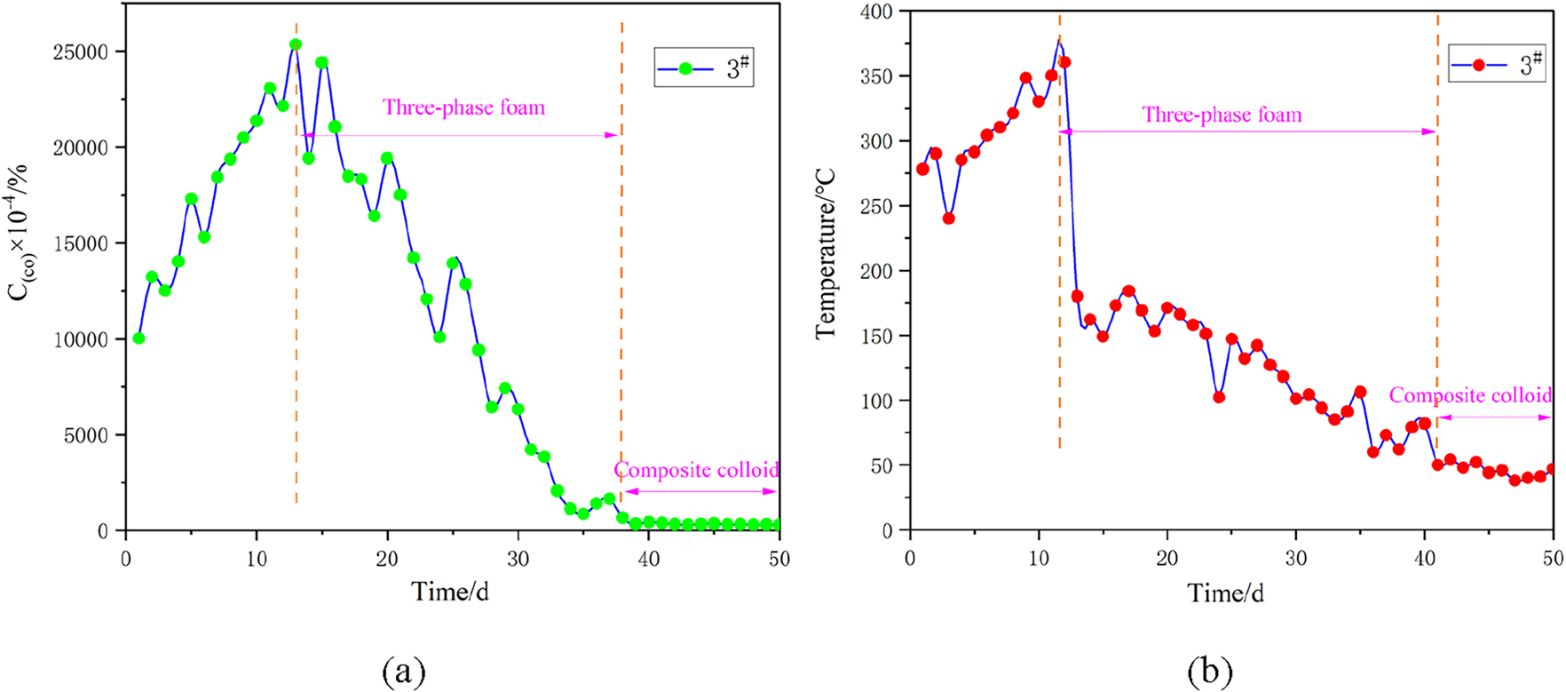
Treatment effect analysis for 3# borehole: (**a**) Variation of carbon monoxide concentration in the borehole with time; (**b**) Variation of temperature in the borehole with time.

The representative 1#, 2# and 3# boreholes are selected for continuous sampling analysis. The analysis mainly focuses on the concentration and temperature of carbon monoxide, the fire indicator gas inside the boreholes. Specifically, a portable sampling pump is used to extract indicator gas from the borehole, and a meteorological chromatograph is adopted for subsequent detection. The temperature of the borehole is directly detected by a thermocouple. The sampled data are subjected to statistical analysis, and the results are displayed in Figs. 20, 21 and 22. The curves of carbon monoxide in the boreholes reveal that after borehole construction, fractures in the overlying surrounding rock of the coal body are connected, which brings about an increase in air leakage channels and accelerates the formation of the “chimney effect”. This phenomenon aggravates the spontaneous combustion trend of the outcrop coal seam. The carbon monoxide concentrations in 1#, 2# and 3# boreholes all witness an upward trend, evidenced by the rising temperatures inside the boreholes. When the spontaneous combustion risk of the outcrop fire has a potential to rise, the fire prevention and extinguishing system is immediately constructed, and three-phase foam is injected. With these measures taken, the temperatures of the boreholes drop sharply and present an obvious downward trend, while the carbon monoxide concentrations lag and rebound to a certain extent. The reason behind this phenomenon is that when three-phase foam acts on the spontaneously combusting coal body, the heat of the high-temperature coal body is taken away instantaneously, and the temperature of the borehole drops rapidly. However, due to the large scope of the fire area and the considerable need for heat exchange, the fire area has not been completely extinguished. Moreover, under the impact of fractures, the spontaneous combustion indicator gas experiences no apparent changes. After thirty days of pouring three-phase foam, the outcrop fire area has been under effective control. Subsequently, composite colloid is injected into the fractures, efficiently filling the fractures in the overlying strata of the outcrop coal seam. The above treatment measures succeed in effectively controlling the fire indicator gas and the borehole temperature, holding back the re-combustion of the fire area.

**Figure 23:**
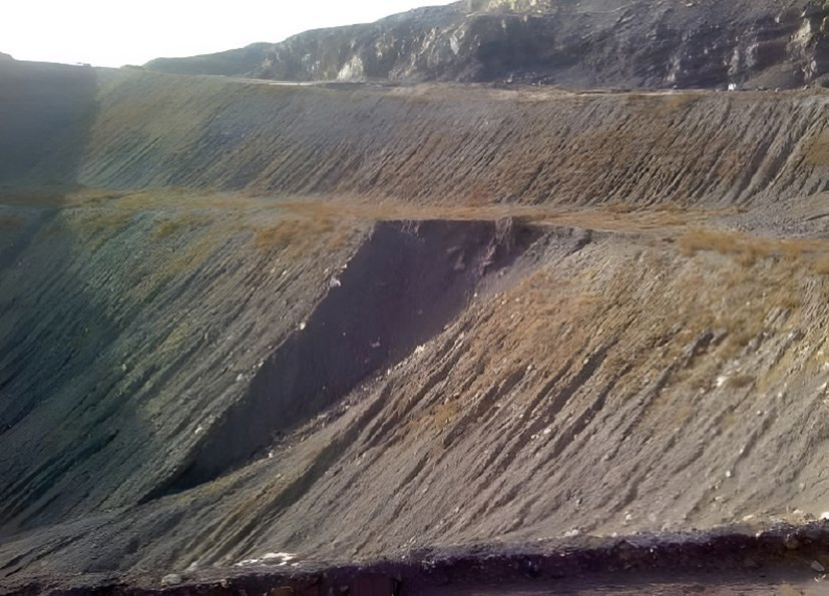
Ecological restoration after fire area treatment.

To test the treatment effectiveness of the outcrop fire area and evaluate the impact of the outcrop fire area on the adjacent 010,203 working face and to eliminate the threat of underground gas explosions caused by hidden danger in the outcrop fire area, samples are taken from the adjacent goaf and gas drainage boreholes after the mining of the working face. The results reveal that neither spontaneous combustion indicator gas nor high-temperature areas are detected, and the southern outcrop fire area has been extinguished, which will not pose a threat to safe mining of the working face. After the treatment is completed, grass seeds are sown in the loess backfill area to restore ecology (Fig. 23).

## Conclusions

Through the detection and treatment of the coal seam outcrop fire area, this project provides a new method for coal seam outcrop fire control and has broad application prospects. The methods and results are summarized as follows:

1. Infrared thermal imaging is only applicable to the detection of high-temperature anomaly areas on the shallow surface. An integrated use of the spontaneous potential method and drilling detection can accurately delineate the range of the outcrop fire area and lay a foundation for subsequent fire area management.
2. The application of comprehensive fire prevention and extinguishing technologies, such as three-phase foam filling fractures for fire extinguishing and cooling, colloid injection for plugging air leakage channels, and loess backfilling for re-combustion prevention, can effectively remove the threats of outcrop fire areas and ensure the safe production of coal mines.

## Data Availability

The data presented in this study are available on request from the corresponding author.
